# Expression and distribution of CD151 as a partner of alpha6 integrin in male germ cells

**DOI:** 10.1038/s41598-020-61334-2

**Published:** 2020-03-09

**Authors:** J. Jankovicova, M. Frolikova, V. Palenikova, E. Valaskova, J. Cerny, P. Secova, M. Bartokova, L. Horovska, P. Manaskova-Postlerova, J. Antalikova, K. Komrskova

**Affiliations:** 1 Laboratory of Reproductive Physiology, Institute of Animal Biochemistry and Genetics, Centre of Biosciences, Slovak Academy of Sciences, Dubravska cesta 9, 845 05 Bratislava, Slovak Republic; 2 Laboratory of Reproductive Biology, Institute of Biotechnology, Czech Academy of Sciences, BIOCEV, Prumyslova 595, 252 50 Vestec, Czech Republic; 3 Department of Biochemistry, Faculty of Science, Charles University, Hlavova 8, 128 40 Prague 2, Czech Republic; 4 Laboratory of Structural Bioinformatics of Proteins, Institute of Biotechnology, Czech Academy of Sciences, BIOCEV, Prumyslova 595, 252 50 Vestec, Czech Republic; 5 Department of Veterinary Sciences, Faculty of Agrobiology, Food and Natural Resources, University of Life Sciences Prague, Kamycka 129, 165 00 Prague 6, Czech Republic; 6 Department of Zoology, Faculty of Science, Charles University, Vinicna 7, 128 44 Prague 2, Czech Republic

**Keywords:** Cell biology, Germline development

## Abstract

The physiological importance of CD151 tetraspanin is known from somatic cells and its outside-in signalling through integrins was described. In male germ cells, two tetraspanins, CD9 and CD81, are involved in sperm-egg membrane fusion, and similarly to integrins, they occupy characteristic regions. We report here on a newly discovered presence of CD151 in sperm, and present its expression and distribution during spermatogenesis and sperm transition during the acrosome reaction. We traced CD151 gene and protein expression in testicular cell subpopulations, with strong enrichment in spermatogonia and spermatids. The testicular and epididymal localization pattern is designated to the sperm head primary fusion site called the equatorial segment and when compared to the acrosome vesicle status, CD151 was located into the inner acrosomal membrane overlying the nucleus. Moreover, we show CD151 interaction with α6 integrin subunit, which forms a dimer with β4 as a part of cis-protein interactions within sperm prior to gamete fusion. We used mammalian species with distinct sperm morphology and sperm maturation such as mouse and bull and compared the results with human. In conclusion, the delivered findings characterise CD151 as a novel sperm tetraspanin network member and provide knowledge on its physiology in male germ cells.

## Introduction

CD151 belongs to the tetraspanin superfamily, these proteins participate in many biological processes such as growth control, intracellular signalling, cell adhesion, migration, motility^1–4^ and they also take place in pathogenesis of some human diseases^5,6^. Tetraspanins create a scaffolding multiprotein network in the membrane that anchors other proteins into specific domains with precise functions and moreover, they are indispensable players in the mammalian fertilization process. Tetraspanin CD9 expressed on mouse eggs is considered as an essential molecule for successful gamete fusion^7–9^ as well as tetraspanin CD81 which is also involved in fertilization^10,11^. Our previous studies concerning species-specific traits of CD9 and CD81 in mouse, bull and porcine gametes during their functional maturation and fertilization, suggested the active participation of tetraspanins in these events^11–14^. With the ability of tetraspanins being part of signalling cascades involving cytoskeleton^5,15,16^ they also play a crucial role in the organization of functional membrane protein cluster domains called a tetraspanin web, which were also described in oocyte and recently in sperm. The integral part of the tetraspanin web are integrins and the presence of α3β1, α6β1 and α6β4 integrins was shown in sperm^17–20^. Importantly, in somatic cells, tetraspanin CD151 was found to play a key role in regulating the adhesion strengthening of integrin α6β1^21^. CD151 has previously been linked with human gamete fusion^22^, associates specifically with α3, α6 and α7 integrins^1,3,23^ and links α3β1 and α6β1 integrins to other tetraspanins, including CD9^24–27^. Based on that knowledge we propose that CD151 could be involved as a part of known multimolecular complexes remodelling sperm membrane through integrins^28,29^. Remodelling of the acrosomal region of the sperm head during acrosome reaction (AR) is a crucial step enabling sperm to become fusion competent. During AR, a dramatic reorganization of sperm head membranes occurs including fusion of plasma and outer acrosomal membranes, in order to release the acrosomal content leaving the sperm head surface covered with inner acrosomal membrane (IAM) exposing new proteins^30–33^. During this event, many of the membrane proteins originally locked in the acrosome relocate to the plasma membrane and different sperm head compartments, mainly to the equatorial segment. This re-location process was previously describe for proteins that are key players of gamete fusion such as Izumo1^30,34^, CD46 and integrins^32^ or tetraspanin CD81 and CD9^13,35^. Structural modelling of CD9 and CD81 interaction in human sperm^13^ suggested the possible role for CD81 as a regulator of dynamic changes in sperm plasma membrane, whereas CD9 appeared to be involved in web stabilization and mediation of *trans* protein binding upon sperm-egg interactions^13^. Hereby, we present data showing CD151 expression and distribution in male germ cells of mouse, bovine and human and it is a first report that CD151 protein is part of the tetraspanin network on sperm. We also provide evidence that CD151 is bound to the α6 integrin subunit in mouse sperm, and propose a structural model of this interaction. The overall presented findings extend the knowledge of sperm specific tetraspanins members and their role in crucial events preceding fertilization in mammals.

## Results

In order to address CD151 role in male germ cells in a complex manner we targeted three selected species, such as mouse, bull and human. We combined a pool of methods suited to individual species as they display distinct sperm morphology as well as reproductive strategies including sperm behaviour prior to fertilization.

### Mouse

**Figure 1:**
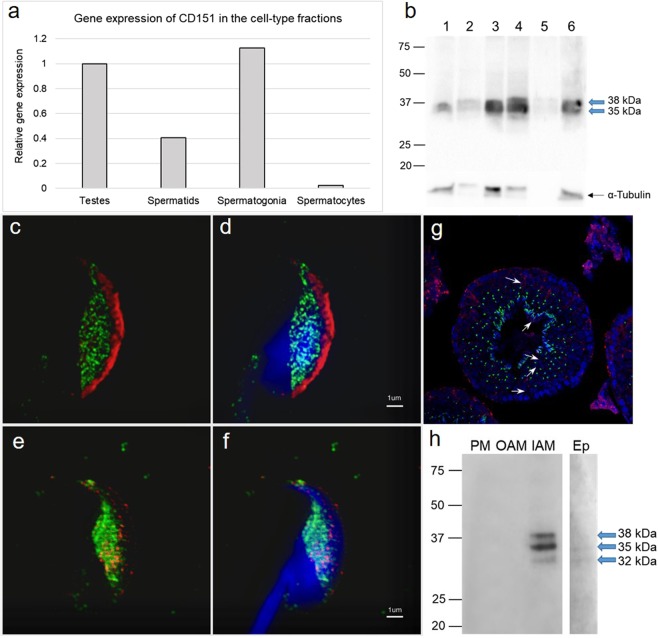
Detection of CD151 in the mouse testis and spermatozoa. (**a**) Gene expression of CD151 in the cell-type fractions from mouse testis. Cq value of the gene is normalized by reference gene Rps2. Numbers > 1 are considered as strongly expressed in the individual cell-types; Spermatids – round spermatids; Spermatocytes – primary spermatocytes. **(b)** Detection of CD151 protein in elutriation fractions: 1 and 2 – round spermatids, 3 and 4 – spermatogonia, 5 – primary spermatocytes, 6 – spermatogonia, Sertoli and Leydig cells; antibody detected protein bands of ∼32 and 35 kDa (blue arrows). The protein concentration in testicular cell samples was checked by α-tubulin detection. **(c–f)** Localization of CD151 in sperm head detected by super-resolution SIM capturing **(c**,**d)** acrosome-intact epididymal and **(e,f)** acrosome-reacted. CD151 (green) is localized in membrane overlying equatorial segment. **(c,d)** PNA-lectin (red) was used for detection of the acrosomal status. **(d**,**f)** Nucleus is visualized by Dapi (blue). Scale bar represents 1 µm. **(g)** Detection of CD151 (red) on cryo-sections of mouse testes (white arrows point at different germinal cell populations with positive CD151). Nucleus is visualized with Hoechst (blue). Transgenic C57BL/6J^Acr3-EGFP^ were used as mouse line with EGFP (green) labelled acrosome in spermatids of relevant stages and sperm. **(h)** Western blot immunodetection of CD151 in sperm membrane fractions (PM – plasma membrane; OAM – outer acrosomal membrane + tails; IAM – inner acrosomal membrane + residual heads) and extract form epididymal sperm (Ep); antibody detected bands of ∼32, 35, and 38 kDa in inner acrosomal membrane fraction, and bands of ∼32 and 35 kDa in whole extracts from mouse epididymal sperm (blue arrows), which likely represent glycosylated isoforms of CD151 protein.

To assess the expression of CD151 in mouse germinal cells, we targeted at first testicular cells using the elutriation technique and evaluated *CD151* gene expression in individual cell fractions. To determine enrichment of individual fractions by relevant sperm cell type we performed q-RT-PCR and defined each fraction by specific gene markers. We identified the germinal cells using gene markers for spermatogonia, primary spermatocytes, round spermatids, round/elongating spermatids, Leydig and Sertoli cells and the data are summarized in the Supplementary Table 1. After characterization of the elutriation fractions, we analysed the expression of *CD151* gene investigated by q-RT-PCR. mRNA expression was mainly detected in the population of spermatogonia and round spermatids (Fig. 1a). Furthermore, we used specified elutriation fractions for protein immunoblotting and detected CD151 as two dominant bands of ∼32 and 35 kDa with the evident highest expression in spermatogonia and spermatids (Fig. 1b). In order to detect a precise localization of CD151 we performed super-resolution Structure Illumination Microscopy (SIM) using sperm from *cauda epididymis* to monitor the protein distribution in the sperm cells prior to ejaculation. Based on the fluorescent detection of intact sperm, the CD151 is located in the acrosomal region of the head, specifically in its distinct equatorial segment, and it is absent from the apical acrosomal area (Fig. 1c,d). In sperm after the acrosome reaction it remains located in the equatorial segment and therefore occupies the primary sperm-egg fusion region (Fig. 1e,f). In order to correlate findings of gene and protein expression during spermatogenesis (Fig. 1a,b), with testicular localization, we used immunohistochemical staining for CD151 on testicular cryo-sections using transgenic C57BL/6J^Acr3-EGFP^ mice. These mice express a green fluorescent protein in the acrosome^36^, which is a potent tool to monitor spermiogenesis and distinguish round spermatids from other cell populations (Fig. 1g). In accordance with previous findings, CD151 was strongly localized in spermatogonia as well as spermatocytes and spermatids. The distal acrosomal region was becoming visible in late stages of spermatid development and in sperm (Fig. 1g). The localization of CD151 in epididymal sperm was also confirmed by immunoblotting (Fig. 1h) and CD151 was detected as two bands with molecular masses of ∼32 and 35 kDa. Based on the high-resolution photographs, we could observe the localization of CD151 in the IAM and to confirm this localization we also performed a membrane fractionation of the epididymal sperm. We detected the CD151 in the IAM fraction as three specific bands of ∼32, 35 and 38 kDa, which correspond with both elutriation fractions and whole epididymal extract. The three described protein bands may possibly indicate post-translational modifications, particularly glycosylation, of the CD151 protein in mouse sperm.

### Bull

**Figure 2:**
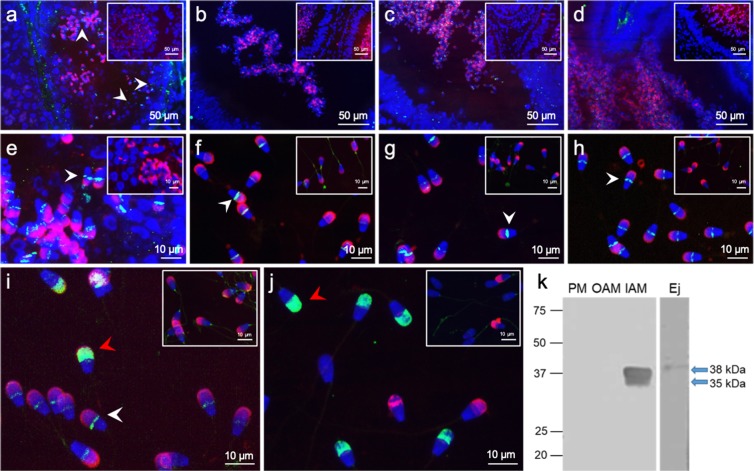
Detection of CD151 in the bull testis, epididymis and spermatozoa. Detection of CD151 (green) on cryo-sections of **(a)** testes (white arrows point at different germinal cell populations with positive CD151) **(b)** *caput epididymis*, **(c)** *corpus epididymis*, **(d)** *cauda epididymis*; **(e)** in spermatozoa within the testes; in the isolated spermatozoa from individual parts of *epididymis* **(f)** *caput*, **(g)** *corpus*, **(h)** *cauda*; **(i)** in ejaculated spermatozoa, **(j)** in spermatozoa after acrosome reaction. Sperm acrosomes are labelled by PNA lectin (red); nuclear DNA was stained by DAPI (blue). Rabbit IgG isotype control is situated in the top right corner. White arrows show CD151 in the equatorial segment of spermatozoa, red arrows points to CD151 in IAM of spermatozoa. **(k)** Western blot immunodetection of CD151 in protein extract of sperm membrane fractions (PM – plasma membrane; OAM – outer acrosomal membrane + tails; IAM – inner acrosomal membrane + residual heads) and extract form ejaculated sperm (Ej). Antibody detected bands of ∼35 and 38 kDa in IAM fraction, and band of ∼38 kDa in the whole extract of bull ejaculated sperm (blue arrows).

CD151 was strongly visible in late spermatids and testicular sperm as a clear thin line in the equatorial segment (Fig. 2a,e). There was also a low fluorescent signal detectable in other populations of germ cells in their early development (Fig. 2a). The presence of CD151 was further detected on sperm within the *caput*, *corpus* and *cauda epididymis* in tissue sections (Fig. 2b,c). The weakest signal was obtained in dense clusters of sperm from the *cauda epididymis*, probably due to a poorly accessible epitope for the antibody within the mass of sperm in the tubule lumen. Clearly visible and strong labelling of the equatorial segment was observed in isolated sperm not only from caput, but also from the corpus and cauda (Fig. 2f–h). On sperm with a detached acrosome a strong signal of CD151 spread over the whole sperm head was detected. The positive signal of CD151 antibody was visible on more than 70% of evaluated sperm. Localisation of CD151 on ejaculated bull spermatozoa (Fig. 2i) showed to be the same as in epididymal spermatozoa. This expression pattern of CD151 was also observed in sperm head of intact freshly ejaculated and capacitated sperm. Additionally, in a portion of sperm, those with “loosened” acrosomes, a weak signal over the whole sperm head was visible (Fig. 2i). On the surface of acrosome-reacted sperm with uncovered inner acrosomal membrane, fluorescent signal over the anterior region of sperm head was observed (Fig. 2j). CD151 was detectable in this region already after 15 min induction of acrosome reaction and the portion of CD151 positive sperm increased with continued induction (Fig. S1).

Localization of CD151 in the equatorial segment and in the IAM of ejaculated spermatozoa was also supported by immunoblotting analyses of protein extracts from sperm membrane fractions. In bull sperm, we detected double band of ∼35 and 38 kDa exclusively in the inner acrosomal membrane fraction enriched with sperm heads. In the whole sperm extract, the band of ∼38 kDa was only found (Fig. 2k).

### Human

**Figure 3:**
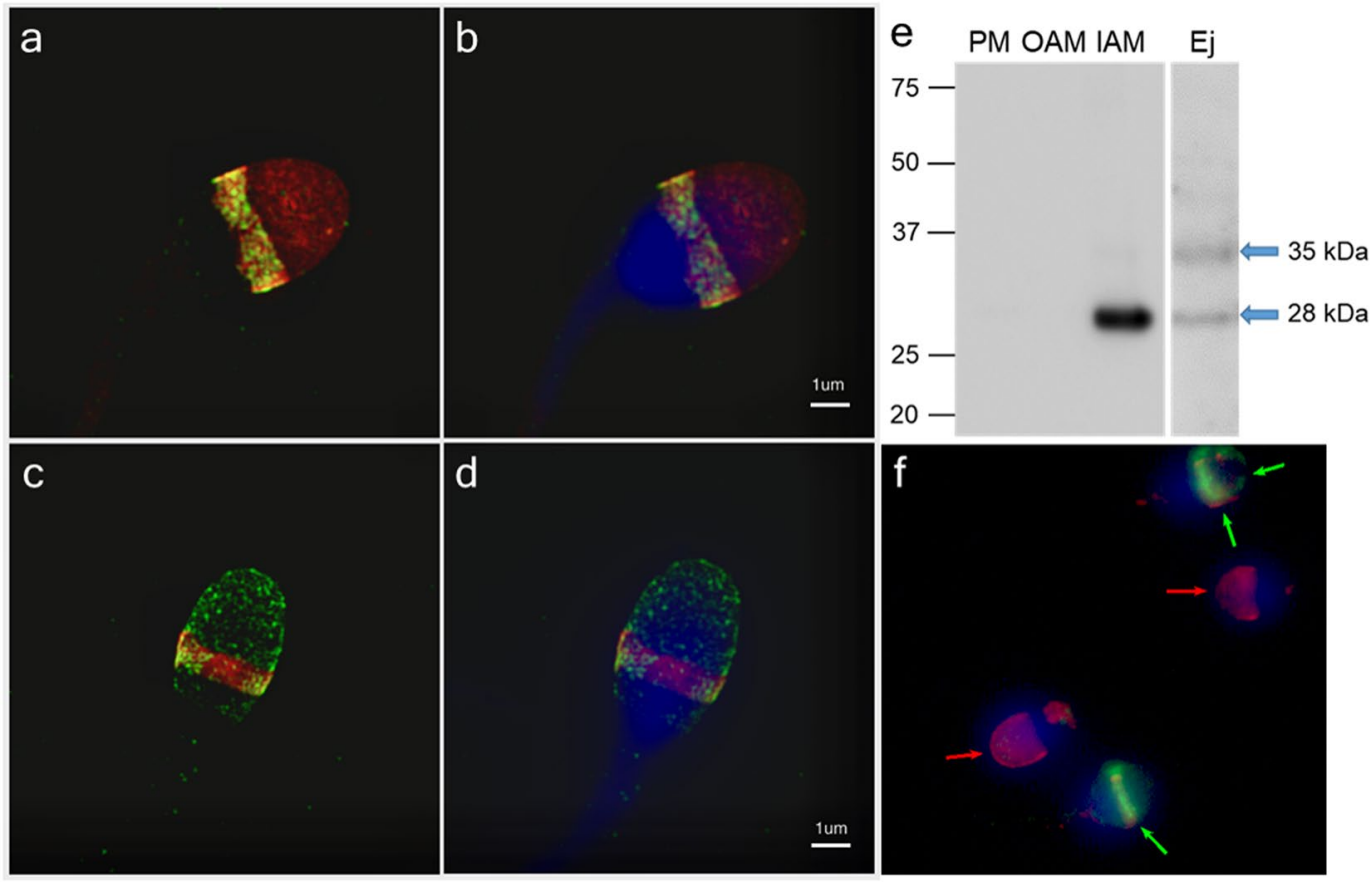
Detection of CD151 on human sperm. (**a–d**) Localization of CD151 detected by super-resolution SIM in **(a,b)** ejaculated acrosome-intact and **(c,d)** acrosome-reacted human sperm head. CD151 (green) is localized in membrane overlying equatorial segment. Scale bar represents 1 μm. **(f)** Detection of CD151 (green) of human intact and acrosome-reacted spermatozoa in unfixed sperm suspension shows the absence of green signal in acrosome intact ejaculated cells (red arrows indicate acrosome intact sperm without antibody reaction); green arrows show positive antibody staining on CD151 (green) in equatorial segment and whole IAM. **(a–f)** PNA-lectin (red) labels the sperm acrosome. DAPI (blue) stains the nucleus. **(e)** Western blot immunodetection of CD151 in sperm membrane fractions (PM – plasma membrane; OAM – outer acrosomal membrane + tails; IAM – inner acrosomal membrane + residual heads) and extract form epididymal sperm (Ep); antibody detected bands of ∼28 and 35 kDa in whole extracts from human ejaculated sperm and strong band of ∼28 kDa in the inner acrosomal membrane fraction (blue arrows).

To reveal a precise localization of CD151 in human sperm, we used super-resolution Structure Illumination Microscopy (SIM). Based on the fluorescent detection of acrosome-intact ejaculated sperm, the CD151 is located in the acrosomal region of the head, specifically in its distinct equatorial segment, and it is absent from the apical acrosomal area (Fig. 3a,b). In sperm after the acrosome reaction it remains located in the equatorial segment and moreover it appears in the acrosomal cap area (Fig. 2c,d). We immunodetected a strong 28-kDa band in the fraction enriched in the inner acrosomal membrane proteins corresponding with the CD151 protein. In the extract from human ejaculated sperm, two protein bands of ∼28 and 35 kDa were found (Fig. 3e; blue arrows). The calculated molecular mass of CD151 is ∼28 kDa, but since one glycosylation site in the protein has been reported^37^, the other bands may easily represent CD151 isoforms with different glycosylation, however, the un-glycosylated form was found only in human spermatozoa (Fig. 3e).

Other detected protein bands with higher molecular weights may indicate different glycosylated isoforms of CD151 protein in sperm. Similarly, two isoforms (28 and 32 kDa) of CD151 has been immunoprecipitated from platelets^38^, and the 32 kDa form has been described in osteosarcoma cell lines^39^. Distinct molecular mass isoforms of CD151 detected in spermatozoa of all investigated mammalian species (32, 35 and 38 kDa) demonstrate that the protein glycosylation can differ among cell types and species^40^.

### Interaction of CD151 with α6 integrin

**Figure 4:**
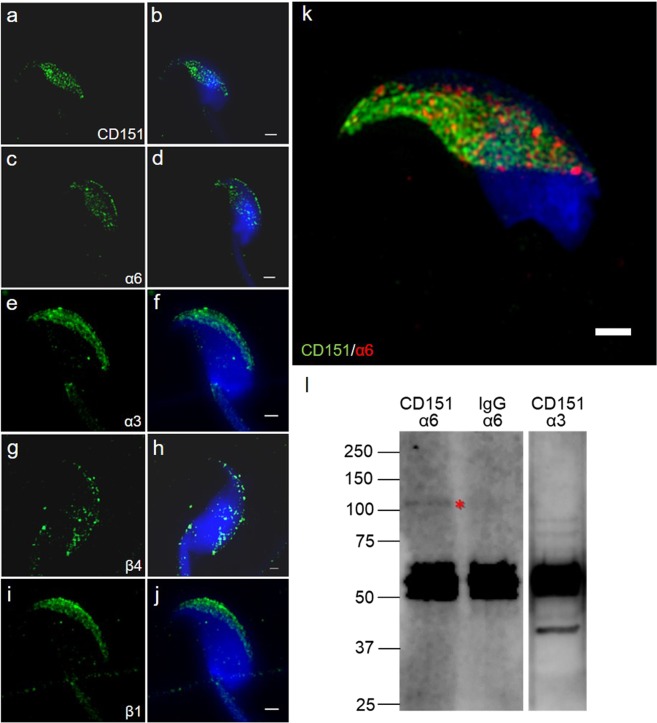
Localization of CD151 and integrin subunits α6 and β4 in mouse epididymal spermatozoa and their interaction. Localization of **(a,b)** CD151 (green), **(c,d)** α6 integrin (green), **(e,f)** α3 integrin (green), **(g,h)** β4 integrin (green), **(i,j)** β1 integrin (green) revealed by super-resolution SIM capturing. CD151 and α6, β4 integrin subunits are localized in the membrane overlying the equatorial segment of the acrosome. On the other hand, α3 and β1 integrin localization is to apical acrosome. Therefore CD151, α6 and β4 are localized to the different compartments then α3 and β1 integrin subunits. **(b,d,e,f,j)** Nucleus is visualized by DAPI (blue). **(k)** Showing the co-localization pattern of CD151 (green) and α6 integrin subunit (red), which is positive in the equatorial segment area. Scale bar represents 1 µm. **(l)** Co-immunoprecipitation of CD151 with α6 integrin (first lane) and α3 integrin (third lane); asterisk indicates detection of α6 integrin band (∼120 kDa; blue arrow) in the CD151 immunoprecipitate of mouse epididymal sperm lysates. Negative control, immunoprecipitation with rabbit IgG followed by a detection with α6 integrin antibody, is shown in the second lane; other bands probably represent heavy chain of immunoglobulin and non-specific interaction of antibody.

By immunofluorescent labelling using super-resolution SIM capturing we detected the same localization, for CD151 tetraspanin as well as α6 and β4 integrin subunits, which is restricted to the equatorial segment of acrosome-intact epididymal spermatozoa in mouse (Figs. 4a–d,g,h). This observation was confirmed using the co-localization staining assay for CD151 and α6 integrin (Fig. 4k). In order to target a possible interaction between these two proteins, we performed co-immunoprecipitation analysis using mouse sperm lysate obtained by weak detergent (CHAPS). Using the specific antibody we detected α6 integrin in molecular mass of ∼120 kDa in CD151 immunoprecipitate (Fig. 4l; asterisk). On the other hand, α3 and β1 integrin subunits (Figs. 4e,f,i,j) localized in distinct area of the sperm head than CD151 was found. Additionally, anti-α3 integrin antibody did not recognize any relevant protein band corresponding to α3 integrin (∼120 kDa) in CD151 immunoprecipitate (Fig. 4l, third lane).

**Figure 5:**
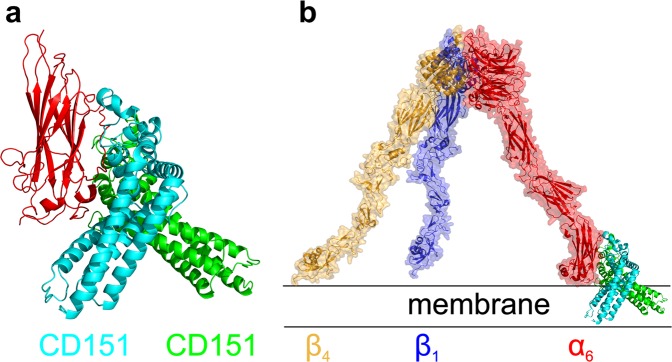
CD151 interactions predicted by docking to the C-terminal domain of the extracellular part of integrin α6. **(a)** Two predicted binding modes of the α6 C-terminal domain (red) with the CD151 involving its extracellular part. The model shows CD151 in a predicted higher affinity mode (green) as well as in a lower affinity mode (cyan). **(b)** The predicted positions of CD151 models after a structure superposition to the previously derived models of the α6/β1 (red/blue) and α6/β4 (red/orange) integrin complexes. The model shows that the transmembrane regions of the CD151 are tilted with respect to the membrane plane and the biologically relevant interaction might involve the CD151 and α6 intergrin domain in its more extended conformation.

In order to identify probable interacting interfaces between the CD151 and integrins we have performed a flexible side chain protein-protein docking of the CD151 model to the isolated N- and C-terminal extracellular domains. The results show that in most cases the docking procedure predicts integrin domain interactions with the transmembrane part of CD151 which is however immersed in the membrane and not available for interaction in the biologically relevant arrangement. The only predicted interaction involving the extracellular part of CD151 corresponds to interaction with the C-terminal extracellular domain of integrin α6. Two potential binding modes are predicted by docking (Fig. 5a). Structural superposition of the C-terminal domain to the model of extracellular part of the α6β1 integrin complex (Fig. 5b) reveals that the transmembrane regions of CD151 are tilted with respect to the membrane plane.

## Discussion

In mammalian sperm, the equatorial region is considered as the initiation site of gamete fusion^36^. Sperm released to *rete testis* have functional domains characterized by a specific protein and lipid composition which is ensured by membrane compartmentalization. During the acrosome exocytosis, a vesiculation of membranes occurs and although the equatorial segment is not directly involved in these processes and it seems to be rigid in terms of its structure, tremendous dynamic changes were documented on biochemical level. Several proteins, such as Izumo1^41^, protein DE^42^, equatorin^43^, tetraspanin CD9^13,44^ and other important proteins for sperm-egg interaction or fusion relocalize from the apical acrosome to the equatorial segment during AR. We report here on the presence of another tetraspanin protein CD151 in the equatorial segment and moreover, on its localization in the IAM that occurs just after the AR is completed. Together with the existence of α6β4 integrin in the same localization and detected interaction of CD151 with α6 integrin subunit we propose the existence of a transmembranous protein system responsible for stabilization of multimolecular complexes arrangement which is assembled to the equatorial region and ensures its ability to interact with the egg. Up to now, in every co-expressing somatic cells analysed to date, CD151 makes a fundamental contribution to integrin-dependent motility, invasion, morphology, adhesion and/or signalling. Moreover, CD151 knockdown has a novel and unexpected dysregulating effect on the model of α6 integrin diffusion^45^. It will be interesting to addressed this somatic cell based CD151 physiology in sperm in the future.

There is a remarkable consistence in our results among studied species when we describe CD151 protein expression in germ cells during their development and consequent maturation stages including the protein detection after the AR. From our previous finding on tetraspanins CD9 and CD81, their characteristic pattern to some extent differed between mouse, bull and human and retained its species specific uniqueness^12,13,35^. However, in mouse and human, CD9 location in the equatorial segment after the AR stands out^13^, similarly to our current results, CD151 is destined to this sperm primary fusogenic region. Based on the knowledge that members of tetraspanin family such as CD9, CD37, CD53, CD63, CD81, CD82, CD151 and others, contribute to the structural organization of the plasma membrane by forming microdomain structures, influencing cell fusion and regulating cell motility^46^, not all of them can interact directly with integrins. It is of relevance that namely CD81 and CD151 are reported to be a crosslink mediating indirect interaction of integrins with other tetraspanins^24,47^. These membrane cis protein interactions are of importance for “outside-in” signalization, reported from the somatic cells^48,49^ as well as playing their role within female gametes^10,14,22^. However, CD151 is known to participate with the α6β1 integrin in the formation of a functional complex involved in human sperm-egg fusion^22^. The existence of the CD151 tetraspanin network in the equatorial segment of sperm and their interaction with the α6 subunit of presented α6β4/β1 integrin suggest a possibility of trans-interaction of both sperm and egg protein networks during sperm-egg membrane fusion. This is emphasized by the previously reported interaction between CD151 and CD9 tetraspanins in human oocyte with α3 integrin (CD49C) in spermatozoa^50^. Because the CD151 is most probably localized in the membrane environment, offering only the relatively small extracellular part to an interaction, the predicted N-terminal integrin domain contacts would correspond to an integrin interaction in its closed, folded state. The predicted CD151 interaction with the C-terminal extracellular domain of an integrin would then probably correspond to a near membrane interaction with the activated, extended form of an integrin. The tilted arrangement of the transmembrane regions of the CD151 with respect to the membrane plane as predicted by the docking might suggest that the α6 integrin domain interacts with the CD151 in its more extended conformation. However, a more detailed description of the CD151 conformation state or details of its binding interface would require more demanding theoretical as well as experimental study.

In our previous study, we showed a presence of both α6β1 and α6β4 integrin heterodimers on sperm^20^. However, the α6 subunit preferentially creates the α6β4 heterodimer in the presence of both β4 and β1 subunits^23,51,52^. The expression and behaviour of α6β4 as well as its interaction with CD151 is described in hemidesmosome and cancer cells^25,53^ where CD151 plays a role in integrin trafficking and subcellular distribution^54,55^. Moreover, in hemidesmosome, CD151 strengthens the adhesion complex of α3β1 with α6β4 and plectin^56^, which in sperm surrounds the nucleus^57^. Based on the knowledge that CD151 and α6β4, but not α3β1 integrin share the same location in the membrane overlaying the equatorial segment, which defines the cytosolic space above the sperm nucleus, it is possible that CD151 could be involved via α6β4 and plectin in equatorial domain stability during the acrosome reaction preceding the sperm-egg membrane fusion. It is also of relevance, that the β4 integrin subunit is structurally different from the other known β subunits as a result of its unusually large cytoplasmic domain with an ability to affect cytoskeleton organization^2,4,58^ including Rac1 protein and induce its activation^6^. It is of interest, that in sperm, Rac1 was recently shown to play a key role in capacitation and AR^3^ and participates in actin remodelling in the apical acrosome region during these events. Based on this fact we could speculate that CD151 in cooperation with α6β4 could even participate in regulating actin cytoskeleton dynamics in sperm.

It is also likely that previously reported cross-linking of CD46 with the β1 integrin subunit in somatic cells^59^ is of relevance with relocation of CD46 into the equatorial segment of the sperm^32^ where it can associate through direct interaction with β1 integrin and indirectly with tetraspanins such as CD151 via α3, α6 integrin subunit^59,60^. Even though the primary CD151 location is indisputably in the equatorial region, it is interesting to point out the observed dynamic behaviour of this protein during the AR. We detected CD151 in acrosome-reacted sperm and also in the anterior IAM, which defines the acroplaxome overlaying the nucleus. One can speculate, if due to massive relocation of many crucial proteins involved in sperm-egg membrane recognition and fusion, the CD151 tetraspanin network could also be actively remodelled during AR from the posterior part of the acrosome equatorial segment to its apical part. We hypothesize that the change in CD151 expression pattern after the acrosomal exocytosis could possibly be also due to unavailability of its epitope for an antibody binding in acrosome-intact sperm and CD151 could be expressed on the IAM prior to AR.

In conclusion, with knowledge that CD151, as a member of the tetraspanin superfamily, possess specific biological characteristics, described in somatic cells, our finding that this protein is expressed in male germ cells during spermatogenesis and remains localized in sperm during epididymal transport and ejaculation that opens up a wide range of further possible investigations. Moreover, we deliver evidence that the specific localization of CD151 is in the primary fusion segment of the sperm head and become surface exposed only after the acrosome reaction. We also report on extended CD151 pattern into the apical part of the head covered at the stage of gamete fusion by inner acrosome membrane. These results are true for all the studied species such as mouse, bull and human, despite the previously reported species-specific differences for many other sperm proteins including tetraspanins. We also bring findings that CD151 is bound to the α6 integrin subunit in mouse sperm, and based on structural modelling we propose that the α6 integrin domain interacts with the CD151 in its more extended conformation of this interaction. We believe that the overall presented findings extend the knowledge of tetraspanin role in crucial events foregoing the fertilization in mammals.

## Material and Methods

All chemical reagents were obtained from Sigma Aldrich (St. Louis, MO, USA) unless otherwise noted. We confirm that all methods were performed in accordance with the relevant guidelines and regulations.

### Animals

C57BL/6J mice were purchased from the Animal Resources Centre or produced by the animal breeding facilities of the Institute of Biotechnology. The mice were housed in the IMG animal facilities, Institute of Molecular Genetics of Czech Academy of Science, Prague, and food and water were supplied ad libitum. The mice were healthy 10–12 weeks old animals with no sign of stress or discomfort. Transgenic reporter C57BL/6J^Acr3-EGFP^ mice expressing green fluorescent protein in the acrosome of developing spermatids and mature spermatozoa^36^ were used for testicular histology sections. These reporter transgenic male mice were generated in the Transgenic Unit of the Czech Center for Phenogenomics at the Institute of Molecular Genetics CAS and they are property of the Laboratory of Reproductive Biology, IBT CAS, Vestec, Czech Republic. The mice were housed in animal facilities of the Institute of Molecular Genetics of Czech Academy of Science, Prague, and food and water were supplied ad libitum. All the animal procedures and all the experimental protocols were approved by the Animal Welfare Committee of the Czech Academy of Sciences (Animal Ethics Number 66866/2015-MZE-17214, 18 December 2015).

Freshly ejaculated or frozen-thawed bovine sperm were obtained from bulls (Bos taurus) of Slovak Breeding Services, Inc., Luzianky, Slovak Republic. The bull epididymides were obtained at a local slaughterhouse (Mala Maca, Slovakia). The study was carried out according to the Council Directive 98/58/EC, Council Regulation (EC) No. 1099/2009, Regulation (EU) 2016/1012, Slovak National Council No. 39/2007 and guidelines of the Slovak legislation (directive 432/2012 Z. z.).

### Tissues

#### Elutriation

Elutriation protocol was performed according^61^. The elutriation was done in PBS at 4 °C on a Centrifuge J26XP with elutriation rotor JE-5.0 (Beckman Coulter, Indianapolis, IN). The precise conditions are described in Table S1. The cells were collected into 50 ml tubes on ice. Cells in each tube were pelleted by centrifugation (400 × *g*, 15 min, 4 °C) and resuspended in Tri-reagent (Sigma-Aldrich). The total RNA was isolated according to manufacturer’s instructions and stored at 70 °C. The protein fractions were precipitated by acetone, dissolved in reducing sample solution (2% SDS in Tris-HCl, pH 6.8; 5% mercaptoethanol) with 0.5% Protease Inhibitor Cocktail, incubated for 30 min at 4 °C and boiled for 5 min.

The bull tissues segments (testes and epididymides) were preserved by TissueTek (Sakura Finetek, Alphen aan den Rijn, NL) and frozen in liquid nitrogen. The frozen sections (5-µm) were cut using a Leica Cryocut 1800 cryostat (Leica Microsystems, Wetzlar, Germany), fixed for 5 min in a cold acetone-ethanol mixture (1:1), air-dried and washed in PBS.

#### Reverse transcription and Real time quantitative PCR (q-RT-PCR)

Total RNA was isolated from testicular fractions prepared by elutriation and testes samples using TRI Reagent (Sigma-Aldrich). Firstly, RNA extracts (2 μg) were treated with DNase I (1 U/μL, Fermentas, Hanover, MA) in presence of DNase I buffer 10× (Thermo Scientific) with MgCl_2_ for 30 min at 37 °C and EDTA (Fermentas) was added for 10 min at 65 °C. The reverse transcription reaction contained 5x reaction Buffer (Fermantas), Riboblock Inhibitor (20 U/μL, Sigma-Aldrich), Universal RNA Spike II (0.005 ng/μl, TATAA biocenter, Sweden), 10 mM dNTP Mix (Thermo Scientific), oligo(dt)18 (Thermo Scientific) mixed 1:1 with Random primers (Thermo Scientific) and M-MuLV RevertAid transcriptase (200 U/μL, Fermentas), and run for 60 min at 42 °C followed by 10 min at 70 °C to generate cDNA.

For q-RT-PCR cDNA (10 ng/µl), two times Maxima SYBR Green qPCR Master Mix (Thermo Scientific), reverse and forward primer (1 μM, Generi Biotech, Hradec Kralove, Czech Republic) and nuclease free water were used.

The *Ribosomal protein S2* (*Rps2*) gene was used as the reference gene. Specific gene markers for germinal cells and somatic cells were selected to determine elutriation fractions (the enrichment of individual elutriation fractions is listed in the Supplementary Table 1).

### Spermatozoa

Mouse spermatozoa were obtained from cauda epididymis, released into M2 medium, incubated for 15 min at 37 °C in 5% CO_2_, washed in PBS and centrifuged at 300 × *g* for 10 min twice at room temperature (RT).

Bull spermatozoa were obtained from epididymides divided into three segments: the caput, corpus and cauda. The segments of caput and corpus were cut into small pieces, incubated in 10 ml of PBS for 15 min at 37 °C. Spermatozoa from ducts of cauda were blown with syringe. Spermatozoa were washed with PBS and centrifuged at 200 × *g* for 10 min at RT.

Freshly ejaculated spermatozoa were separated from seminal plasma by centrifugation at 200 × *g* for 10 min at RT and washed with PBS. The pellets of frozen-thawed spermatozoa were washed in PBS at 200 × *g* for 10 min at RT.

Human ejaculates were obtained from Centrum for assisted reproduction Gennet (Prague, Czech Republic) with the informed consent of healthy donors and in accordance with the Institutes’ Human Ethics Committee guidelines, from men after 3-4 days of sexual abstinence. After liquefaction, the spermatozoa was separated from seminal plasma by centrifugation gradient (55%/80%) of SupraSperm System (Origio, Måløv, Denmark) and centrifuged at 300 × *g* for 20 min at 37 °C. Biological materials and experimental protocols were approved by Ethics committee of the General University Hospital, Prague, number 617/17 S-IV.

### *In vitro* capacitation and induction of the acrosome reaction

Mouse sperm from the distal regions of the cauda epididymis were released into a 200 μL droplet of M2-fertilizing medium (M7167) under paraffin oil and pre-tempered at 37 °C in 5% CO_2_. Released sperm were assessed for motility and viability under a light inverted microscope with a thermostatically controlled stage at 37 °C. Sperm (5 × 10^6^/mL) were left freely to capacitate for 90 min in 100 μL M2 medium under paraffin oil. The acrosomal reaction was induced by Calcium Ionophore (CaI, A 23187) at a final concentration of 5 μM for 90 min at 37 °C in 5% CO_2_.

Bull sperm were re-suspended in a commercially supplied TL medium for bovine sperm cell capacitation (Minitube, Celadice, Slovak Republic), supplemented with 6 mg/mL BSA, 0.02 mol/L Na pyruvate and 0.5 mg/mL gentamycin, to a final concentration of 10^7^ cells/mL and capacitated for 4 h at 39 °C in 5% CO_2_. Acrosome reaction was induced by 10 µMol/L CaI A23 187 for 1 h under the same conditions.

Human sperm was capacitated in 0.5 ml of Sperm Preparation Medium (Origio, Måløv, Denmark) for 2 h at 37 °C under 5% CO_2_. The acrosomal reaction was inducted by CaI A 23187 in a final concentration of 10 µM for 1 h at 37 °C in 5% CO_2_.

### Immunolabelling of spermatozoa and tissues

For super-resolution microscopy spermatozoa were fixed at a high precision cover glasses with cold acetone-ethanol for 5–8 min, blocked with Super Block® Blocking Buffer (Thermo Scientific, Rockford, IL, USA) for 30 min at RT and treated with primary antibody or combination of two antibodies in case of double staining, overnight at 4 °C. Secondary antibody for 1 h at RT were applied. The intactness of spermatozoa acrosomes was assessed by Peanut agglutinin (PNA)-Alexa568 (1:500). The nuclear DNA of cells was stained by Hoechst (1:200). After washing, 90% glycerol with 5% anti-fade-N-propyl gallate as a mounting medium was used. For SIM visualisation, following antibodies were used: primary antibodies rabbit polyclonal anti- CD151 antibody (ab125363, Abcam, antibodies, Cambridge, UK) diluted 1:100 in PBS, rabbit polyclonal anti-β1 integrin antibody (sc- 8978, Santa Cruz Biotechnology, Inc., Dallas, TX, USA) 1:10, rabbit polyclonal anti-α3 integrin antibody (H-43, Santa Cruz Biotechnology, Inc., Dallas, TX, USA) 1:10, mouse monoclonal anti-α6 integrin antibody (F-6, Santa Cruz Biotechnology, Inc., Dallas, TX, USA) 1:10, rabbit polyclonal anti-β4 integrin antibody (bs-4115R, Bioss antibodies, Woburn, MA, USA) 1:10; secondary antibodies Alexa fluor 488 goat anti-rabbit IgG and Alexa fluor 488 goat anti- mouse IgG or Alexa fluor 568 donkey anti- mouse IgG (Molecular Probes, Eugene, OR, USA) diluted 1:300 in PBS. SIM super-resolution images were obtained by Zeiss Elyra PS.1 inverted microscope at Laboratory of confocal and fluorescent microscopy of Faculty of Science (Charles University, Prague, Czech Republic). Representative results are shown.

Cryo-sections of testicular tissue of Transgenic C57BL/6J^Acr3-EGFP^ mice expressing green fluorescent protein in the acrosome of developing spermatids and mature spermatozoa were used for immunolabeling. Tissue was fixed for 10 min with cold acetone-methanol (1:1) and dried. After 30 min of blocking with Super Block® Blocking Buffer (Thermo Scientific, Rockford, IL, USA), primary anti-CD151 antibody (ab125363, Abcam, antibodies) in concentration 1:50 in PBS was applied for 2 hours, followed by secondary antibody Alexa fluoro 568 goat anti-rabbit IgG (Molecular Probes, Eugene, OR, USA) (1:300) for 1 h at room temperature. In the end, the slides were mounted into a Vectashield mounting medium with DAPI (Vector Lab., Burlingame, CA, USA). Images were obtained with high-end confocal microscope Carl Zeiss LSM 880 NLO (Imaging Methods Core Facility at BIOCEV, Vestec, Czech Republic). An open source software Fiji^62^ was used for further image processing.

Bull tissue sections and sperm smears were fixed for 5 min by cold acetone-methanol (1:1) and dried. After blocking, anti-CD151 antibody or rabbit IgG isotype control (1–2 µg/mL) was applied. Goat anti-rabbit IgG-fluorescein (FITC) conjugated secondary antibody (1:300) (Vector laboratories, Burlingame, CA, USA) was applied, followed by PNA-TRITC staining. The nuclear DNA of cells was stained by Vectashield mounting medium with DAPI (Vector laboratories). Immunostaining was evaluated under a Leica DM5500 B epifluorescence microscope at 400× and 1000× magnifications. The fluorescence images were recorded with a Leica DFC340 FX digital camera and processed using Leica Advanced Fluorescence software. Representative results are shown.

### Isolation of sperm plasma and acrosomal membranes

Fractionation was made according to Somanath and Gandhi (2004). Fresh bull ejaculate (10 ml) was diluted 1:2 with Krebs Ringer Bicarbonate (KRB) medium: 0.7 mM Na_2_HPO_4_ + 0.49 mM MgCl_2_ + 4.56 mM KCl + 0.1198 M NaCl + 0.0013 M NaH_2_PO_4_ + 2.37 mM fructose + 0.0149 M NaHCO_3_ (pH 7–7.6)). Human and mouse sperm were prepared as above and diluted 1:2 with KRB medium (devoid of BSA, containing 2.37 mM/l glucose). Diluted semen of bull, mouse and human were layered on solution of 1.3 M sucrose with 0.9% NaCl and centrifuged for 30 min at 2000 × *g* at 4 °C. Pellet of sperm was resuspended in 0.15 M NaCl with 5 mM HEPES (pH 7.0), layered on solution of 1.3 M sucrose with 0.9% NaCl and centrifuged for 20 min at 34000 × *g* at 4 °C. The pellets were resuspended in 0.15 M NaCl with 5 mM HEPES (pH7.0) supplemented with protease inhibitors and sonicated 1 × 5 s and 3 × 2 s for mouse and 20 × 10 s intervals for human (Ultrasonic, 80 Amplitude microns power), and 10 × 10 s intervals (Soniprep 150, 30 Amplitude microns power) for bull. Membranes from homogenate were separated by discontinuous sucrose gradient consisting of 1.75 M sucrose and 1.3 M sucrose (1:1) and centrifugation for 3 h at 95000 × *g* at 4 °C. The plasma membrane fraction was at the interface between the sample and 1.3 M sucrose; acrosomal membrane fraction was the interface between 1.3 M/1.75 M sucrose. Pellet contained IAM and remaining equatorial segments closely associated with the sperm heads. The fractions of plasma membrane and acrosomal membrane fraction were diluted with PBS and pelleted by centrifugation for 30 min at 120000 × *g* at 4 °C. The fraction of IAM was washed by PBS and centrifuged at 3500 × *g* for 10 min at 4 °C. Subsequently the separate membrane fractions were solubilized with 1% (v/v) Triton X-100 for 1 h at 4 °C. Proteins from human and mouse fractions were precipitated by acetone, all protein samples were then incubated at 100 °C for 5 min in reducing sample solution.

### SDS-PAGE electrophoresis and Western blot analysis

Pellets of human and bull ejaculated and mouse epididymal spermatozoa were dissolved in reducing sample solutions for 30 min at 4 °C and subsequently boiled for 5 min at 100 °C.

The protein extracts from elutriation fractions of mice testes, membrane fractions and spermatozoa of mouse, bull and human were separated by 12% SDS-PAGE and transferred onto nitrocellulose membrane (Advantec Toyo Kaisha Ltd., Tokyo, Japan). The molecular weights of the separated proteins were estimated using Dual Color Prestained Protein Standards (Bio-Rad, Hercules, CA, USA). After blocking, the membranes were incubated with anti-CD151 antibody (ab125363, Abcam) overnight at 4 °C, followed by incubation with secondary antibody: goat anti-rabbit IgG conjugated to horseradish peroxidase, or goat anti-rabbit IgG conjugated to alkaline phosphatase for 1 h at RT. Antibody reaction was visualized with SuperSignal West Pico Chemiluminescent Substrate (Thermo Scientific, Rockford, IL, USA) for HRP-conjugated antibody (mouse and human) or with NBT (4-nitroblue tetrazolium chloride) and BCIP (5-bromo-4-chloro-3-indolyl-phosphate) solution for antibody conjugated with alkaline phosphatase (bull).

### Co-immunoprecipitation of CD151 and α6 integrin

Mouse sperm suspension (5 × 10^7^) was lysed in 100 µl of Tris buffer (20 mM Tris-HCl, 137 mM NaCl, pH 8) with 1% CHAPS at 4 °C for 1 h. Lysates were centrifuged at 10,000 *g* for 5 min at 4 °C. Then supernatant was incubated with polyclonal antibody anti-CD151 (ab125363, Abcam) in final amount 2 µg per sample overnight at 4 °C in rotator. As a control, rabbit IgG (Abcam) in the same concentration as antibody was used. Twenty µl of washed Agarose Protein A/G (Thermo Scientific) were added and incubated for 2 h at RT. Precipitates bound to Protein A/G were washed twice with lysis buffer for 5 min at 4 °C in rotator. This procedure was repeated for three times. Co-immunoprecipitated complexes were eluted from Protein A/G agarose by incubation in reducing sample buffer for 5 min at 100 °C. After electrophoretic separation in 10% polyacrylamide gel and transfer into PVDF membrane, the CD151 immunoprecipate was incubated with mouse antibody against α6 integrin (F-6, Santa Cruz Biotechnology, Inc., Dallas, TX, USA) and rabbit antibody α3 integrin (H-43, Santa Cruz Biotechnology, Inc.) diluted 1:100 in PBS overnight at 4 °C. After washing and incubation with secondary anti-mouse or anti-rabbit antibody conjugated with HRP (Bio-Rad) diluted 1:3000, antibody reaction was visualised by SuperSignal West Femto Chemiluminescent Substrate (Thermo Scientific).

### Molecular modelling of CD151/integrin interactions

We have used the previously prepared all atom models of extracellular parts of integrins α3, α6, β1, and β4^20^ built using a local copy of the I-TASSER^63^ service, based on the annotation of protein sequences as defined by UniProt^64^ under accession codes Q62470, Q61739, P09055, and A2A863. The homology model of the murine CD151 was built using I-TASSER based on the O35566 UniProt sequence similarly to our previous tetraspanin models^13^. The flexible side chain protein-protein docking of the CD151 model to the N- and C-terminal extracellular domains of integrins was performed using a local copy of the ClusPro server^65,66^. Analysis of modelling results, structure superposition and graphical visualization was performed using the PyMOL program^67^ version 2.1.0.

## Supplementary information

Table S1, Figure S1.
