# Supplementary material for: Expression and distribution of CD151 as a partner of alpha6 integrin in male germ cells
Source: Sci Rep. 2020 Mar 9;10:4374. doi: 10.1038/s41598-020-61334-2 (PMC7062741; doi:10.1038/s41598-020-61334-2)
Supplement: Supplementary file 1 — Table S1, Figure S1. [file 41598_2020_61334_MOESM1_ESM.pdf]

SUPPLEMENTARY INFORMATION

Expression and distribution of CD151 as a partner of alpha6 integrin in male germ cells.

Jankovicova J [1x], Frolikova M [2x], Palenikova V [2,3], Valaskova E [2], Cerny J [4], Secova P [1], Bartokova M [1], Horovska L [1], Manaskova-Postlerova P [2,5], Antalikova J [1*] and Komrskova K [2,6*]

[1]Laboratory of Reproductive Physiology, Institute of Animal Biochemistry and Genetics, Centre of Biosciences, Slovak Academy of Sciences, Dubravska cesta 9, 845 05 Bratislava, Slovak Republic; [2]Laboratory of Reproductive Biology, Institute of Biotechnology, Czech Academy of Sciences, BIOCEV, Prumyslova 595, 252 50 Vestec, Czech Republic; [3]Department of Biochemistry, Faculty of Science, Charles University, Hlavova 8, 128 40 Prague 2, Czech Republic; [4]Laboratory of Structural Bioinformatics of Proteins, Institute of Biotechnology, Czech Academy of Sciences, BIOCEV, Prumyslova 595, 252 50 Vestec, Czech Republic; [5]Department of Veterinary Sciences, Faculty of Agrobiology, Food and Natural Resources, University of Life Sciences Prague, Kamycka 129, 165 00 Prague 6, Czech Republic; [6]Department of Zoology, Faculty of Science, Charles University, Vinicna 7, 128 44 Prague 2, Czech Republic

Supplementary Table S1. Markers of elutriation. Characterization of the cell population of testicular elutriation fractions using specific gene markers for each population of cells by RT-qPCR. Normalization by *Rps2* housekeeping gene. Data show relativity between cell populations and whole testes; more than 1 is consider as highly enriched by the cell-type.

**Gene Markers**	**Cell populations after elutriation**							**Primary source**
	fraction 1	fraction 2	fraction 3	fraction 4	fraction 5	fraction 6	testes	
C-kit	0.399	1.325	**3.807**	**4.306**	0.677	**25.474**	1	Spermatogonia
Sycp3	0.356	0.903	1.286	1.665	**1.029**	1.412	1	Primary spermatocytes
Acrv1	**1.520**	**2.244**	1.300	0.571	0.309	0.639	1	Round spermatides
Dbil5	0.943	0.975	0.447	0.239	0.202	0.278	1	Round/elongating spermatides
Cyp11a1	0.008	0.083	0.838	2.452	0.858	**18.124**	1	Leydig cells
Wt1	0.062	0.221	0.224	0.219	0.131	1.114	1	Sertoli cells

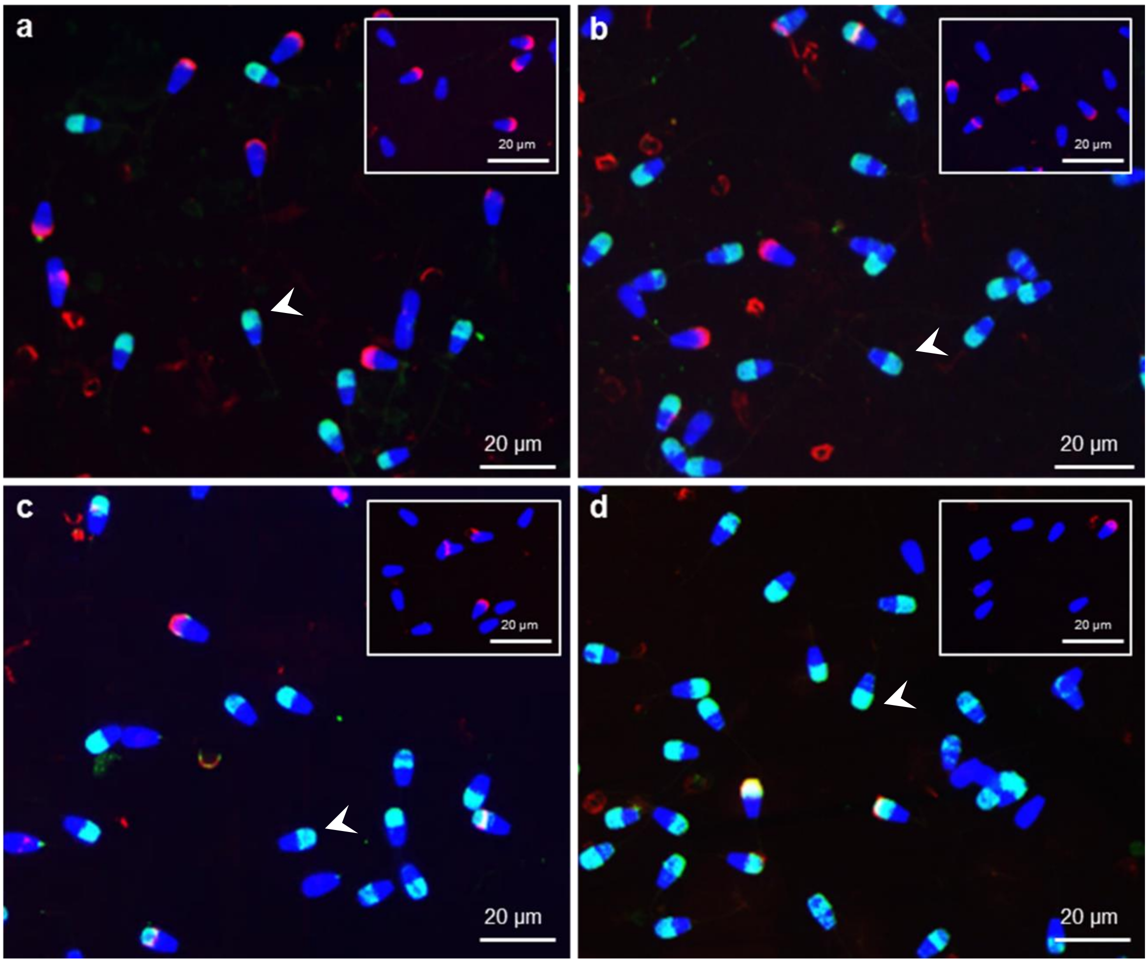

Supplementary Figure S1

Detection of CD151 in the bull spermatozoa during proceeding acrosome reaction. Detection of CD151 (green) on sperm after 15 min **(a)**, 30 min **(b)**, 45 min **(c)**, and 60 min **(d)** induction of acrosome reaction by CaI. Sperm acrosomes are labelled by PNA lectin (red); nuclear DNA was stained by DAPI (blue). Rabbit IgG isotype control is situated in the top right corner. White arrows point to CD151 in the inner acrosomal membrane of spermatozoa.
